# Impact of a wearable-based physical activity and sleep intervention in multimorbidity patients: protocol for a randomized controlled trial

**DOI:** 10.1186/s12877-023-04511-y

**Published:** 2023-12-14

**Authors:** Bernardo Neves, Eduardo D. Haghighi, Hugo V. Pereira, Filipe Costa, João S. Carlos, Daniel Ferreira, Plinio Moreno, Pedro M. Ferreira, Jaime Machado, Breno Goncalves, José Maria Moreira, Francisca Leite, Nuno André da Silva

**Affiliations:** 1 Hospital da Luz Learning Health, Luz Saúde, Lisboa, Portugal; 2 Internal Medicine Department, Hospital da Luz Lisboa, Luz Saúde, Lisboa, Portugal; 3 Centro de Medicina Desportiva, Hospital da Luz Lisboa, Luz Saúde, Lisboa, Portugal; 4 CIDEFES - Centro de Investigação em Desporto, Educacao Fisica, Exercicio e Saude, Universidade Lusofona, Lisboa, Portugal; 5 Value Based Healthcare, Luz Saúde, Lisboa, Portugal; 6 General Practice/Family Medicine Department, Hospital da Luz Lisboa, Luz Saúde, Lisboa, Portugal; 7 Instituto de Sistemas e Robótica (ISR/IST), LARSyS, Instituto Superior Tecnico, Unviersidade de Lisboa, Av. Rovisco Pais 1, 1049-001 Lisboa, Portugal; 8 Heinz College and at the Department of Engineering and Public Policy, Carnegie Mellon University, Pittsburg, USA

**Keywords:** Physical activity, Adequate sleep, Multimorbidity, Older adults, Wearable devices, Behavioral change techniques, Patient Reported Outcome Measures (PROMs)

## Abstract

**Background:**

The benefits of physical activity (PA) and adequate sleep are well documented, and their importance strengthens with the increasing prevalence of chronic diseases and multimorbidity (MM). Interventions to promote physical activity and sleep that use commercial activity trackers may be useful non-pharmacological approaches to managing individual health; however, limited evidence exists on their use to improve physical activity in older adult patients with MM.

**Methods:**

This study aims to measure the effects of behavioral change techniques (BCTs) delivered by a wearable device on physical activity and quality of sleep (QS) in older adult patients with MM. We designed an open-label randomized controlled trial with participants recruited through primary care and a specialist outpatient clinic. Participants must be more than 65 years old, have MM, and have access to smartphones. All eligible participants will receive PA promotion content and will be randomly assigned to wear a smartwatch. The primary outcome will be the participants’ PA measurement at baseline and at six months using the International Physical Activity Questionnaire - Short Form (IPAQ-SF). Secondary outcomes will include changes in the participants’ frailty status, biometric measurements, quality of life, and biopsychosocial assessments. A sample size of 40 participants per arm was calculated to detect group differences, with 50 participants planned to recruit and randomize into each arm.

**Discussion:**

This study aims to contribute to a better understanding of PA patterns and the impact of wearable-based PA interventions in patients with MM. In addition, we aim to contribute to more knowledge about the relationship between PA patterns, Patient Reported Outcomes Measures (PROMs), and healthcare resource utilization in patients with MM. To achieve this, the study will leverage a locally developed PROMs registry and assess data from participants’ medical records, in order to understand the added impact of wearable data and medical information data on predicting PROMs and unplanned hospital admissions.

**Trial registration:**

NCT05777291

**Supplementary Information:**

The online version contains supplementary material available at 10.1186/s12877-023-04511-y.

## Background

Concurrently, the harmful effects of physical inactivity, which refers to the level of Physical Activity (PA) that fails to meet the guidelines, and the benefits of meeting these guidelines are well established [1–3]. PA and exercise, the latter being a planned, structured, repetitive version of physical activity that promotes physical fitness maintenance or development, are key components of lifestyle medicine [1]. The benefits of PA and exercise extend beyond improving mental health and quality of life and contribute to the prevention and treatment of several chronic diseases, including but not limited to obesity, cardiovascular conditions, diabetes, and various types of cancer [4–6].

Lack of sleep has been suggested to play a significant role in the development of major non-communicable diseases (NCDs), which motivates sleep habits to be considered an important modifiable health risk factor and a key component of a healthy lifestyle [7]. Additionally, most manifestations of NCDs worsen with age [8]. As part of lifestyle medicine, further changes in PA and sleep may constitute useful non-pharmacological approaches to managing individual health in older adults [9, 10]. While some aspects of behavior change are still not fully understood, the effectiveness of behavioral change techniques (BCTs) in both altering and sustaining health behaviors, including physical activity and sleep, has been proven, even among the geriatric population [11, 12].

Activity monitoring bracelets, smartwatches, and other wearables may serve as convenient delivery channels for these BCTs and could assist older adult patients in implementing the previously mentioned changes [13, 14]. A recent systematic review showed strong evidence of the positive impact of wearables on increasing physical activity among the general population, although effect on physiological and psychosocial measures was not clear [15]. Despite these findings, limited evidence exists on older adult patients with multimorbidity, both on objectively measured PA as well as on the efficacy of using activity trackers to improve PA [16]. Commercial off-the-shelf activity trackers allow users to self-monitor their daily PA, including the number of steps, type of PA, and amount of sleep. Fitbit (Fitbit Inc, San Francisco, CA, USA) activity trackers have previously been utilized as both measurement and intervention tools, however, it is unclear how they are being integrated into PA intervention studies, and their use in Multimorbidity (MM) patients remains limited [16, 17]. Moreover, in order to access the impact of the PA the patient’s view must be considered. This can be measured using PROMs which are often not considered in these studies [18, 19].

## Methods/Design

### Aims of the study

The main purpose of this study is to measure the effect of the use of BCTs delivered by a wearable device in addition to an intervention to promote PA and quality of sleep (QS) in reported activity by an older adult population with MM. We also aim to assess PA measurements experienced by patients with MM, their relationship with patient-reported outcomes, and adverse clinical outcomes.

### Design and setting of the study

This study will be an open-label randomized control trial, reported in line with the Consolidated Standards of Reporting Trails (CONSORT) statement and checklist and Template for Intervention Description and Replication (TIDieR) checklist [20, 21]. Participants will be recruited through primary care and specialist outpatient clinic at Hospital da Luz Lisboa, a private tertiary hospital in Lisbon, Portugal.

### Inclusion and exclusion criteria

Patients may be included if they are more than 65 years old and have MM. We will use a previously proposed definition of MM as the presence of two or more of the following chronic conditions: hypertension, depression or anxiety, chronic musculoskeletal conditions causing pain or limitation, arthritis and/or rheumatoid arthritis, osteoporosis, asthma, chronic obstructive pulmonary disease (COPD), ischemic heart disease, peripheral artery disease, heart failure, cerebrovascular diseases, chronic stomach or colon conditions, chronic hepatitis, diabetes mellitus, thyroid disorders, any active cancer in the previous five years, chronic kidney disease, chronic urinary conditions, hyperlipidemia, and obesity [22]. In addition, participants must have access to their smartphones. Exclusion criteria will include: patients who are sufficiently physically active, defined with International Physical Activity Questionnaire - Short Form (IPAQ-SF) > 150 min aerobic physical activity per week, an existing absolute contraindication for PA according to the American College of Sports Medicine (acute myocardial infarction within two days, ongoing unstable angina, uncontrolled cardiac arrhythmia with hemodynamic compromise, active endocarditis, symptomatic severe aortic stenosis, decompensated heart failure, acute pulmonary embolism, pulmonary infarction, deep venous thrombosis, acute myocarditis or pericarditis, acute aortic dissection, physical disability that precludes safe and adequate testing), poor comprehension of Portuguese language, disabling neurological disorder (defined as mRankin score $\ge$ 4), severe psychiatric illness, learning disability, dementia and cognitive impairment, registered blind, housebound or resident in a nursing home, non-ambulant, advanced cancer, and scheduled for surgery within five months after the first consultation. Orthopedic or rheumatologic diseases with severe impairment and chronic pain syndromes with inherently reduced mobility will also not be included.

### Study intervention

All Participants who are eligible and consented to participate in the study shall receive PA promotion content in accordance to the Swedish model [23]. This method consists of five core components: (1) person-centered health promotion consultation; (2) a written prescription of physical activity; (3) a prescription guided by evidence-based knowledge on physical activity in the prevention and treatment of health conditions; (4) a follow-up of the written prescription; and (5) collaboration between the healthcare service and physical activity organizers outside healthcare. It is also emphasized that the method should be tailored to the local conditions in healthcare organizations. More specifically, we will recommend PA and provide informative flyers according to the Portuguese National Healthcare Authority and the World Health Organization recommendations for older age people. In addition to PA promotion counseling, all patients will be randomly allocated to either the intervention or the control groups using block randomization generated by a computer-generated random number sequence^1^. The allocation sequence will be generated by a research assistant who will not be involved in the data collection process. Eligible participants will be randomly assigned in a 1:1 ratio to one of the following intervention arms: Arm A (experimental arm), use of the device activity watch (Fitbit Sense), and a sleep monitoring mattress (Withings SA, France - Sleep Analyzer); Arm B (control): non-use of the devices. Participants randomized to wearable device BCT (Arm A) will be provided goal setting, self-monitoring and feedback. On the first visit they will undergo a brief training session where they’ll learn how to effectively use both devices, sync their data, and interpret the feedback they receive. Arm A participants will receive weekly feedback based on their activity and sleep data trough their devices. This might include suggestions for increasing step counts or tips for better sleep hygiene. The study team will provide a communication channel available for participants to address any technical issues or queries they might have regarding device usage. They will be instructed to wear the wearable device all the time (except when charging) and use the mattress while sleeping. Patients who do not use the device for at least 60% of the time will be excluded from the trial. Compliance of divide use will be regularly monitored by the study team.

### Study measures and outcomes

**Fig. 1:**
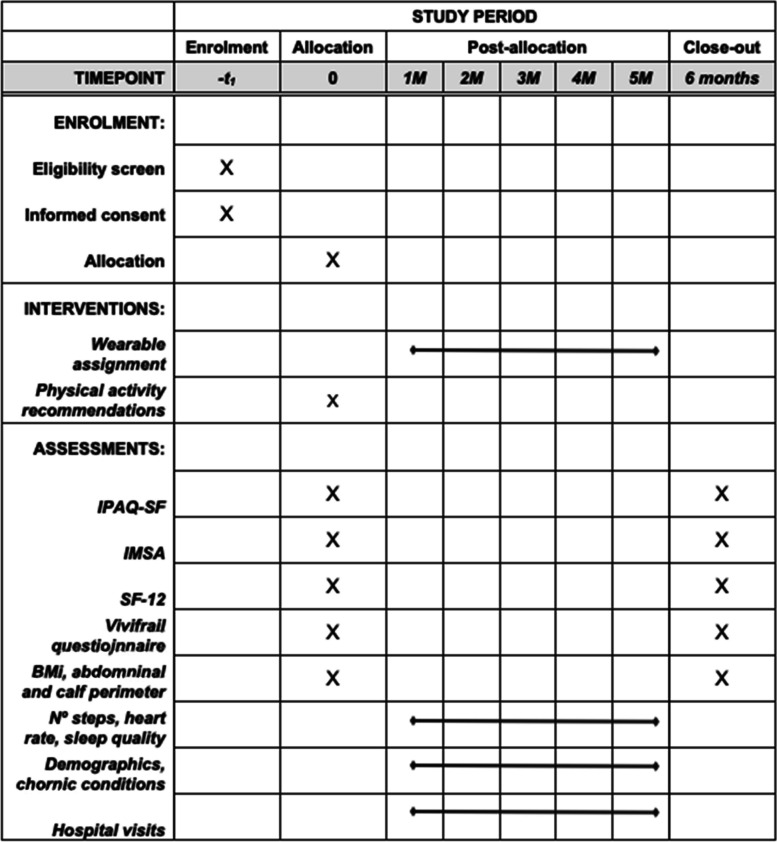
SPIRIT diagram

All participants will have two study visits: baseline and after 6-month follow-up (Fig. 1). Measurements will include self-reported data: IPAQ-SF, INTERMED-Self Assessment (IMSA) and Short Form Health Survey (SF-12); study team measurements: Vivifrail test^2^, Body Mass Index (BMI), abdominal perimeter and calf perimeter measurements; and Electronic Health Records (EHRs) information retrieval (diagnoses, medications, and unplanned health visits before and during the study period). Information on the treatment allocation will not be provided to the data collectors. The data collection forms only contain unique identifier codes assigned to each participant.

The primary outcome will be the participants’ PA measurement assessed at baseline and at 6 months using the IPAQ-SF. Secondary outcomes will include changes in participants’ frailty status, biometric measurements, quality of life, and biopsychosocial assessments at baseline and at 6 months (Table 1).

**Table 1:** Data measurements to be recorded during the study period

	Baseline	Study period	6 month follow-up
**Self-reported**	IPAQ-SF		IPAQ-SF
	IMSA		IMSA
	SF-12		SF-12
**Healthcare professional reported**	Vivifail questionnaire		Vivifail questionnaire
	BMI		BMI
	Abdominal perimeter		Abdominal perimeter
	Calf perimeter		Calf perimeter
**Sensor data**		Number of steps	
		Heart rate	
		Sleep quality	
**EHR data**	Historical clinical data	Unplanned hospital admissions	

### Statistical analysis

A sample size of 40 participants per arm was calculated to be sufficient to detect a group difference, with the alpha set at 0.05, and the power set at 0.80. Protecting against a dropout rate of 20% over the 6-month study duration, 50 participants will be recruited and randomized into each arm (A or B). We will conduct an exploratory analysis of the recorded data. This will be based on descriptive statistics and will be used to characterize the universe of data and identify outliers and general trends. Agreement between IPAQ-SF and wearable activity will be assessed trough Cohen’s kappa. We will use McNemar’s test to compare groups at baseline and 6-month follow-up regarding the study outcomes. In addition, we will assess the impact of wearable data and PROMs in predicting unplanned hospital admissions using classification accuracy, absolute error, and ROC AUC analysis.

## Discussion

We will employ the IPAQ-SF to measure the level of activity in all patients and therefore detect differences between patients who will be using wearables and those who will not. Despite conflicting evidence regarding the correlation with objectively measured PA, this test is highly reported in the literature and has been previously validated in Portuguese. We expect that the results of this study will contribute to better knowledge about the PA patterns of patients with MM as well as the impact of PA intervention in this population, a topic that is underexplored in the literature [16].

In addition, we aim to explore the influence of different PA patterns on other important outcomes such as PROMs and healthcare resource utilization. For this purpose, we will make use of a PROMs registry that has been developed locally for the purpose of data entry. We aim to understand the added impact of wearable data on medical information data for predicting PROMs and unplanned hospital admissions. Data from participants’ medical records will be assessed in the following fields: previous diagnosis, previous hospital episode types and specialties, previous medical procedures, biometrics (weight, height, and BMI), clinical notes, laboratory results, and drugs prescribed or administered in an inpatient setting. The recorded data will be anonymized and stored locally. We aim to understand whether there is any added impact of using this information for future modeling.

### Supplementary Information

**Additional file 1.**

## Data Availability

Not applicable.

## Abbreviations

BCTs: Behaviour change techniques
BMI: Body mass index
COPD: chronic obstructive pulmonary disease
CONSORT: Consolidated Standards of Reporting Trails
EHRs: Electronic Health Records
IMSA: INTERMED-Self Assessment
IPAQ-SF: International Physical Activity Questionnaire - Short Form
MM: Multimorbidity
NCDs: Non-communicable diseases
PA: Physical activity
PROMs: Patient Reported Outcome Measures
QS: Quality of sleep
SF-12: Short Form Health Survey
TIDieR: Template for Intervention Description and Replication

## Footnotes

1 https://www.randomizer.org

2 https://www.vivifrail.com
