# Supplementary material for: Impact of a wearable-based physical activity and sleep intervention in multimorbidity patients: protocol for a randomized controlled trial
Source: BMC Geriatr. 2023 Dec 14;23:853. doi: 10.1186/s12877-023-04511-y (PMC10720080; doi:10.1186/s12877-023-04511-y)
Supplement: Supplementary file 1 — Additional file 1. [file 12877_2023_4511_MOESM1_ESM.pdf]

HOSPITAL DA LUZ
LISBOA

Comissão de Ética para a Saúde

Hospital da Luz

Exmo. Sr. Dr. Bernardo Neves

Medicina Interna

Hospital da Luz

Ref. CES/02/2023/JAG

23 de Fevereiro de 2023

ASSUNTO: ESTUDO “Declaração | Id. 500 | IntelligentCare Study - The impact of a wearable-based intervention in an ambulatory Hospital setting to improve health in multimorbidity through physical activity and sleep” -

A Comissão de Ética para a Saúde do Hospital da Luz, analisou e aprovou o estudo “Declaração | Id. 500 | IntelligentCare Study - The impact of a wearable-based intervention in an ambulatory Hospital setting to improve health in multimorbidity through physical activity and sleep” do qual é V. Exa. Investigador principal.

Pel’ A Comissão de Ética para a Saúde

José Andrade Gomes, Presidente

Hospital da Luz Lisboa • hospitaldaluz.pt/lisboa
Avenida Lusiada, 100 • 1500-650 Lisboa • Portugal
T. +351 217 104 400 • F. +351 217 104 409 • E. geral.lisboa@hospitaldaluz.pt
Hospital da Luz, SA • Capital Social: [illegible] €
Registo da C.R.C. de Lisboa e Contribuinte nº 507 485 [illegible]

LUZ SAÚDE
